# Viral Infections May Be Associated with Henoch–Schönlein Purpura

**DOI:** 10.3390/jcm12020697

**Published:** 2023-01-16

**Authors:** Mariam Nikolaishvili, Ani Pazhava, Vito Di Lernia

**Affiliations:** 1 Faculty of Medicine, Ivane Javakhishvil Tbilisi State University, 0179 Tbilisi, Georgia; 2 American MD Program, Tbilisi State Medical University, 0186 Tbilisi, Georgia; 3 Dermatology Unit, Arcispedale Santa Maria Nuova, Azienda USL-IRCCS di Reggio Emilia, 42123 Reggio Emilia, Italy

**Keywords:** vasculitis, IgA, vessel, virus, SARS-CoV-2, COVID, vaccine, infection, kidney, childhood

## Abstract

Henoch–Schönlein purpura or IgA vasculitis is the most common type of pediatric vasculitis that may affect adults as well. It is classified as a type of small-vessel vasculitis. It can cause cutaneous and systemic symptoms with a minority of patients developing kidney failure. Little is known about the specific pathophysiology of this disorder, except that it is believed to occur in individuals with abnormally glycosylated IgA1. Serum aberrant IgA1 may form large antigen–antibody complexes which, due to a defective clearance, are able to deposit in the small vessels of the skin, kidney, gut, and joints. A variety of factors, including infectious agents, drugs, and vaccines, have been identified as potential triggers. The majority of cases are preceded by upper respiratory tract infections, and seasonal variations suggest a link with many pathogens. The etiologic agent most frequently associated with IgA vasculitis historically have been group A β-hemolytic streptococcus and common respiratory tract viruses. However, during the current coronavirus pandemic, SARS-CoV-2 infection was identified as a main trigger factor. In addition, IgA vasculitis has been observed following COVID-19 immunization. This review provides insights into the state of the art on the relationship between viral infections, viral vaccines, and Henoch–Schönlein purpura.

## 1. Introduction

Henoch–Schönlein purpura (HSP), also known as immunoglobulin A (IgA) vasculitis, is the most common form of systemic vasculitis in children, with a 20.4/100,000 population incidence rate [1]. Most cases occur in children between the ages of 2 and 8 years. Males are affected twice as frequently as females. HSP may infrequently affect adults as well. It is a small-vessel leukocytoclastic vasculitis caused by immune complex deposition, which may manifest as a systemic or single-organ restricted disease [2]. Commonly affected organs include the skin, kidney, gastrointestinal system, and joints [3]. Although there have been significant steps made toward understanding pathogenetic mechanisms, HSP etiology remains largely unknown. This condition would be induced by an abnormal inflammatory process deriving from immune reactions to various antigenic stimuli in genetically predisposed subjects [4]. The first-degree relatives of affected patients are at increased risk of developing this disease. A strong association with HLA class II alleles, specifically HLA-DRB1 alleles, has been identified. Then, peculiar immune complex deposits play a pivotal role in the pathogenesis with the resulting necrosis of the wall of small vessels.

HSP often occurs after bacterial or viral infections and is more frequent in the winter months. The aim of this review is to make a critical appraisal of the possible relationship between viral infections, viral vaccines, and HSP.

## 2. Pathogenesis

The deposition of immune complexes containing IgA in the small vessels of the skin, the renal mesangium, and the additionally affected organs is the defining pathogenic aspect of HSP.

Human IgA displays a large heterogeneity as regards molecular forms and glycosylation [5] with two subclasses that are differentially distributed between the mucosal and circulatory compartments of the immune system. IgA1 and IgA2 are the two isotypes of IgA. IgA1 predominates in serum, while the percentages of IgA2 are higher in secretions. They may be generated in both monomeric and dimeric forms and are both highly glycosylated proteins. Their structure differs by the absence of a 13-amino acid sequence in the hinge region of the IgA2 molecule [6], which gives it a particular resistance against bacterial proteases and may explain the predominance of IgA2 in mucosal secretions.

**Figure 1:**
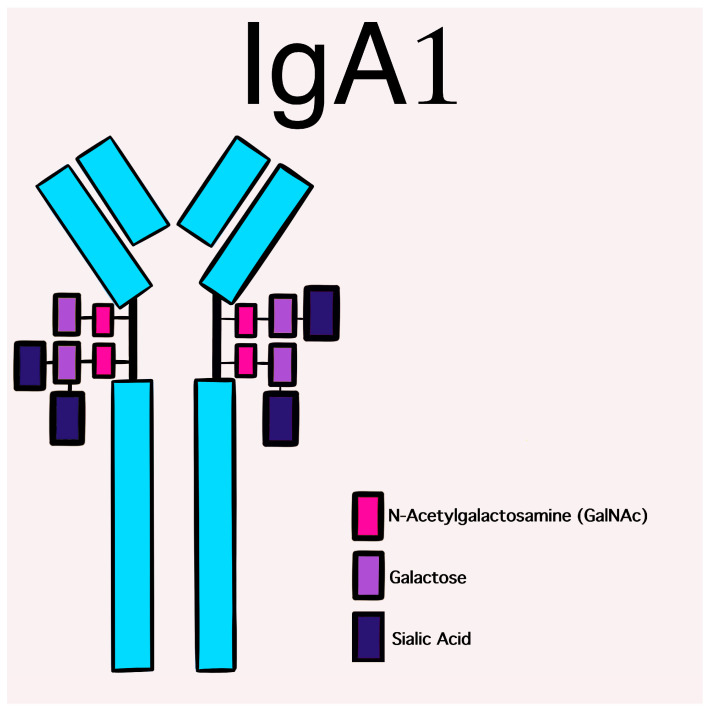
Schematic picture of a normal IgA1 containing GalNAc-galactose disaccharide and its mono- and di-sialylated forms in the hinge region of the heavy chains.

**Figure 2:**
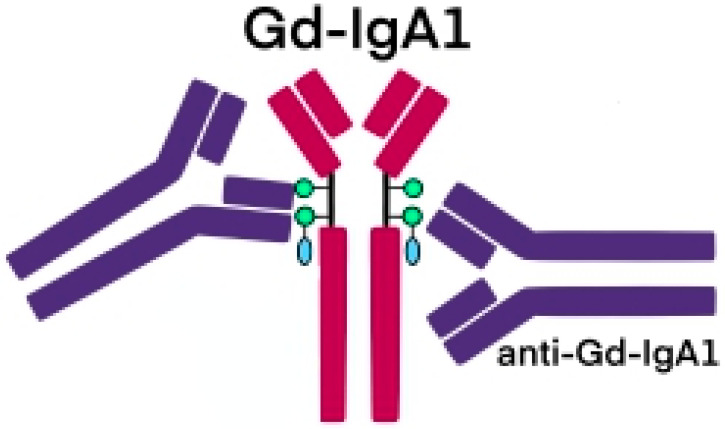
Schematic picture of abnormally glycosylated IgA1 exposing a novel antigenic determinant involving N-acetylgalactosamine (GalNAc), which may be recognized by naturally occurring specific antibodies.

**Figure 3:**
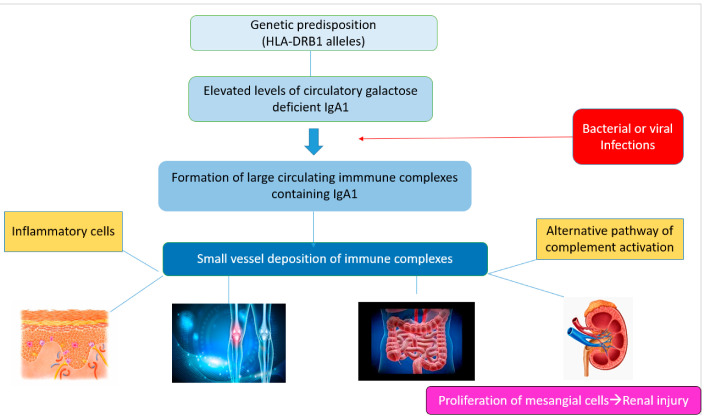
Schematic representation of the main pathogenetic steps of IgA vasculitis.

**Figure 4:**
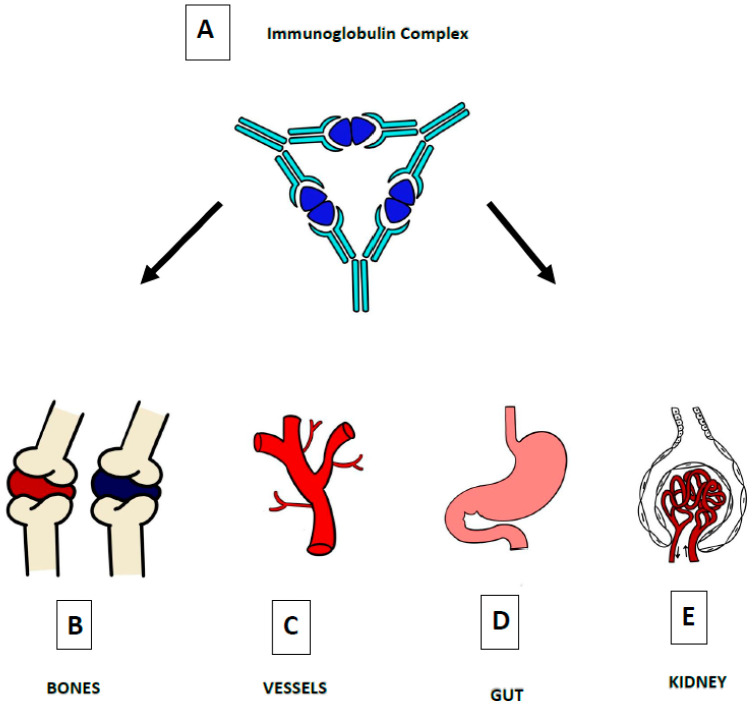
The defining pathogenic mechanism of HSP is due to IgA-containing immune complexes (**A**), which deposit in the small vessels of skin (**C**), joints (**B**), intestine (**D**), and kidneys (**E**).

The glycosylation and size of IgA1 appear to be crucial in promoting IgA1 molecule clearance [7]. Normal interactions between glycosylated IgA1 molecules and the hepatocyte-expressed asialoglycoprotein receptor (ASGP-R) result in the internalization and destruction of these molecules [8]. Patients with HSP, similarly to patients with IgA nephropathy, exhibit poorly galactosylated IgA1 O-glycoforms deficient in galactose and/or sialic acid [9]. However, it appears that an increase in the levels of poorly galactosylated IgA1 O-glycoforms is not sufficient in itself to develop HSP. Indeed, investigations involving the relatives of patients detected similar levels of poorly galactosylated IgA1 O-glycoforms without signs or history of HSP [8]. Consequently, it has been considered that a second, subsequent step may be required for the transition to the full phase of the disease. Aberrantly glycosylated IgA1 molecules expose N-acetylgalactosamine-containing neoepitopes, which may be recognized by glycan-specific IgG or IgA1 antibodies [10] (Figure 1 and Figure 2). The aberrant galactosylated IgA1 O-glycoforms might act either as autoantigens driving the formation of glycan-specific antibodies in genetically prone individuals or as antigens for cross-reactive antimicrobial antibodies. Evidence increasingly suggests that the next step is the formation of large circulating immune complexes prone to deposition in small vessels [11]. Indeed, soluble immune complexes, due to their increased size, are unable to pass through the space of Disse and connect with the asialoglycoprotein receptor (ASGP-R) on hepatocytes. They can pass through the larger fenestrae in the glomerular capillaries that lie directly above the mesangium. By the alternative complement pathway’s activation and the recruitment of inflammatory cells, these deposited complexes cause damage to glomeruli (Figure 3) [10,11,12]. IgA-containing immune complexes are discovered in patients’ serum, as well as the immune complexes containing C3 and IgA in the skin, intestines, and kidneys (Figure 4).

## 3. Environment, Microbial, and Virus Infections

The etiology of HSP seems to be a combination of genetic predisposition, environmental factors, and infectious agents. Its etiology and pathogenesis remain not fully understood, but a number of factors, mainly infectious agents, drugs, and vaccines have been considered as possible triggers. HSP is more frequent in the autumn and winter months and is usually preceded by a wide variety of upper respiratory tract infections. However, community-based outbreaks of HSP have not been reported. Accordingly, there is enough evidence that only susceptible hosts may develop the disease. Indeed, familial clusters of HSP have been indicated, with siblings affected simultaneously or sequentially [13].

In addition to the pathological findings of IgA deposits on small-vessel walls, the occurrence of polymorphonuclear neutrophils infiltration around vessels, the elevation of IgA serum levels, and proinflammatory cytokines during the acute stage suggest that HSP is a specific immune-mediated entity induced by environmental factors, particularly infections [13]. Therefore, HSP is considered a post-infectious immune-mediated small-vessel vasculitis.

Various mechanisms have been proposed to link infections and HSP. Following infections, aberrant IgA1 could be able to recognize the GalNAc-containing (N-acetylgalactosamine-specific) molecules expressed on bacterial or viral structures and form circulating complexes. They would be deposited in the mesangium, inducing the activation of the mesangial cells, which would finally lead to renal damage. According to another pathogenic model, T-cell activation by microbes during respiratory infections could promote increased levels of the transforming growth factor (TGF)-β, which is able to induce an isotype switch of IgA and increased IgA serum levels [13]. Finally, mucosal infections could lead to the upregulation of IL-6 with the possible development of aberrant glycosylation of IgA1 [14].

Of all the pathogens linked to HSP, group A β-hemolytic streptococcus has been the most studied since it may be detected in up to 50% of individuals with acute HSP by serological testing or bacterial cultures [15]. However, multiple bacteria and viruses have been associated with the development of HSP.

### 3.1. Viruses

#### 3.1.1. Severe Acute Respiratory Syndrome Coronavirus 2 (SARS-CoV-2)

The new coronavirus SARS-CoV-2 was recently identified as the etiological agent of coronavirus disease 2019 (COVID-19). SARS-CoV-2 has been associated with the development of HSP as well.

**Figure 5:**
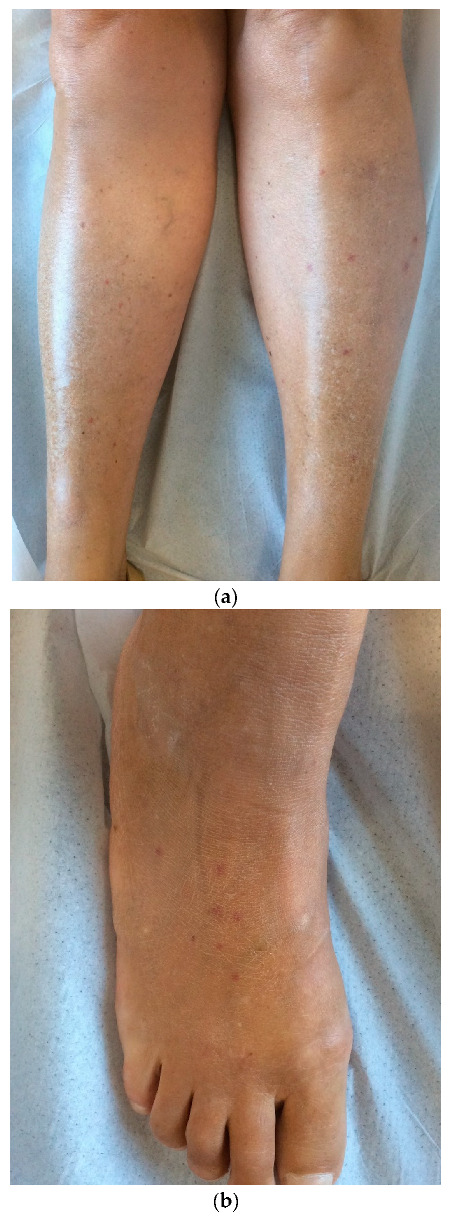
Scattered purpura on lower limbs (**a**) and feet (**b**) of a female patient who tested positive for COVID-19 on antibody testing without additional significant clinical symptoms.

Cases of HSP have first been reported in adults or elderly individuals affected by COVID-19 confirmed with PCR and serologic testing [16,17,18] with high IgA levels in the serum and weak, transitory positivity only for IgA on serologic testing and in patients affected by COVID-related pneumonia [19] (Figure 5). Renal involvement with biopsy-proven focally crescentic and segmentally necrotizing IgA nephropathy [20] or renal function deterioration requiring immunosuppressive therapy have been reported [21]. Interestingly, antineutrophil cytoplasmic antibody (ANCA)-associated vasculitis has also been reported in subjects with previously controlled HSP after COVID-19 infection [22]. A review showed that COVID-19-associated HSP affects mostly adults, and the concomitant presence of other diseases led to a more severe course of vasculitis in older patients developing acute renal failure [23]. From 2021, additional cases of typical HSP occurring with palpable purpura accompanied by abdominal pain, arthralgia, or periarticular swelling were observed in children or adolescents in whom the reverse transcriptase–polymerase chain reaction (RT-PCR) of nasopharyngeal swabs proved positive for SARS-CoV-2 [24,25,26,27]. The onset of HPS was observed also in pediatric or adolescent patients suffering from respiratory symptoms related to COVID-19 [28,29] or developing mild forms of the pediatric multisystem syndrome (PIMS) [30]. Kidney biopsies in pediatric patients with laboratory signs of kidney involvement showed diffuse and segmental mesangial-proliferative glomerulonephritis with the presence of virus-like particles in tubular cells at electron microscopy [31]. No persistence of viral particles has been observed after critical evolution in crescentic glomerulonephritis with sclerosis [31].

A significant decrease in the incidence of HSP was found during the pandemic compared with the pre-COVID era [32]. The observed reduction in incidence is most likely due to the decreased spread of other cold viruses as a result of the precautionary measures (such as wearing masks, restriction of mass gatherings, lockdowns, and quarantine) for reducing virus transmission during the pandemic. These findings suggest that SARS-CoV-2 could be regarded as a less powerful trigger of HSP than other known microbial agents [33].

Organ vasculitis and vascular endothelial injury caused by COVID-19 infection can be explained through different mechanisms, although scientific lines of evidence are lacking. The early seroconversion to IgA in COVID-19 patients and the recognized role of IgA in immune hyperactivation seem to play a dominant role [16,34,35]. SARS-CoV-2 may directly infect endothelial cells or cause an inflammatory response as a result of the infection. Angiotensin-converting enzyme 2 (ACE2) receptors, scavenger receptor B type 1 (SR-B1), and other cell wall receptors facilitating the entry of the virus to the endothelial cells are some of the hypothesized factors involved in viral cell invasion [36].

#### 3.1.2. SARS-CoV-2 Vaccines

Following the start of mass vaccinations as an integral part of the major strategy to reduce COVID-19 numbers worldwide, cases of reactivation and new onset of HSP have been reported [37,38,39,40,41]. All types of coronavirus vaccines were involved, more often after the second dose. The onset of HSP in adult patients generally showed a favorable outcome [41,42,43,44,45], although patients presenting severe glomerulonephritis with a prolonged clinical course were also described [46]. According to a review by Hashizume et al., following COVID vaccination, de novo HSP cases are equally distributed with reactivation cases. The most frequent manifestation is gross hematuria (89.5%), while skin lesions are relatively infrequent [47].

Potential causative factors may be any component of the vaccine, including the nucleoside-modified messenger RNA (mRNA) or the lipid packaging membrane [48]. A reactivation of autoreactive B cells producing IgA following vaccination has been hypothesized [45]. Hence, HSP could be secondary to immune complex formation following the COVID-19 vaccination [48].

Finally, COVID-19-associated HSP is rare. The clinical features of COVID-19-related HSP are not dissimilar from classical HSP, in particular in children [33]. Notably, a relationship between COVID-19 and HSP should be kept in mind. The recognition of this link is crucial to detecting virus infection in asymptomatic COVID-19 virus carriers and consequently limiting the spread of the virus.

#### 3.1.3. Common Respiratory Tract Viruses

The morbidity of HSP shows a noticeable seasonal variation, parallel to some infectious agents. The seasonal distribution shows that HSP more frequently occurs in the winter months, while the lowest onset is in the summer months [49]. Respiratory tract viruses, including parainfluenza virus (PIV), influenza, respiratory syncytial virus (RSV), and adenovirus, are commonly associated with HSP in children [50]. No correlation between the distribution of infectious agents was observed among HSP children with different clinical manifestations. The viral antigens of PIV and RSV have been found in kidney biopsy specimens of children with HSP. A correlation between viral antigens in the kidneys and high urinary microalbumin and 24 h urinary protein has been observed [50].

Influenza vaccines have also been associated with HSP. A small case series consisting of four children developing HSP following influenza vaccinations during the pandemic of influenza A (H1N1) and a review of additional seven patients, including both adults and children, has been reported [51]. The time from vaccination to the onset of the HSP- related symptoms ranged from 1 to 22 days. Five out of eight children reported having a background of immunologically mediated illnesses such as previous HSP, medication eruptions, or food allergies. Although the majority of the patients had a favorable outcome, a 23-year-old man with a 10-year history of HSP and mild renal disease progressed toward end-stage renal disease [52]. Although a temporal relationship between HSP and influenza vaccination was evident in all cases, a causal relationship between them has never been proved in view of the limited number of reports.

#### 3.1.4. Cytomegalovirus

Cytomegalovirus (CMV) has not been reported as a virus associated with HSP onset. However, cytomegalovirus infections may reactivate during HSP. Latent viral infection reactivation has been considered in the setting of immunosuppression. CMV complicating HSP was observed in both children and adults after high-dose steroid therapy [53,54].

#### 3.1.5. Epstein–Barr Virus

The role of Epstein–Barr virus (EBV) in inducing HSP has been extensively investigated in a recent review by Hu and colleagues [55]. The Authors calculated an incidence of 4.2% of EBV-triggered HSP, without clear seasonal variability and a peak prevalence in the 6–10 years age. A significant frequency of abdominal pain was noted. The IgA levels were significantly increased in the patients who developed EBV-triggered HSP.

#### 3.1.6. Hepatitis A, B, C Viruses

HSP has been reported in the context of viral hepatitis or after hepatitis A and B vaccination. In addition, patients with liver cirrhosis may develop HSP as a result of the defective liver metabolism of IgA circulating immune complexes (CICs), resulting in tissue deposition, though this is known to occur without overt vasculitis. The incidence and severity of renal involvement change with age with adult patients showing a higher incidence of nephropathy and progression to renal insufficiency [56,57,58,59].

#### 3.1.7. Measles Virus

The measle virus is not considered a contributing factor associated with HSP. Conversely, measle vaccination has been identified as a risk factor for HSP. The risk of HSP within 12 weeks after immunization was predicted to be significant for measles, mumps, and rubella (MMR) vaccinations, with a threefold increase in the chance of getting HSP [60]. HSP cases were also reported following vaccine delivery in prospective studies undertaken during the MMR immunization campaign. During the Chinese MMR vaccination program, 28 adverse events recorded out of 14.3 million given doses were classified as HSP, with an estimated frequency of 2.1 per million/doses. In conclusion, numbers indicate a very low absolute risk of the disease among children immunized with the MMR vaccination.

#### 3.1.8. Parvovirus B19

Parvovirus B19 has repeatedly been proposed as an etiologic agent in patients with HSP with vasculitis possibly induced by the direct invasion of endothelial cells because of the tissue distribution of the cellular B19 receptor. Single case reports have associated the Parvovirus B19 infection with HSP onset in adults [61,62]. Human parvovirus B19 DNA was identified in dermal and glomerular capillary endothelial cells and surrounding dermal inflammatory cells through in situ hybridization [62]. In addition, human parvovirus B19 DNA, in particular the non-structural protein NS1, has been found in the skin tissues of HSP [63]. However, according to Heegaard and Taaning, parvovirus B 19 is not a common contributing factor associated with pediatric HSP [64].

#### 3.1.9. Varicella Zoster Virus

HPS has rarely been reported following varicella infection in children [65,66,67]. Varicella may reactivate during HSP [68].

## 4. Treatment Modalities

Due to the significant variability in clinical manifestations, pathologic presentation, and long-term outcome, the treatment of HSP is usually guided by clinical presentation.

Recently, evidence-based recommendations were provided not only for the diagnosis but also for the treatment of HSP with the aim to facilitate improvement and uniformity of care [69]. In mild and self-limited cases, supportive care is the first line of treatment. It includes adequate oral hydration, analgesia, and rest. In the early stages of the disease, and if renal function is normal, non-steroidal anti-inflammatory drugs are usually administered for HSP-associated arthritis and arthralgias [70], which are typically non-migratory, transient, and non-destructive, more often involving the ankles and knees. Corticosteroids are widely administered in clinical practice, but a systematic review has shown that they are not able to prevent kidney disease and should not be used prophylactically [69,71]. Corticosteroids are indicated in the presence of mild nephritis or significant complications such as orchitis, cerebral vasculitis, pulmonary hemorrhage, or other severe organ- or life-threatening vasculitis manifestations [69]. Close monitoring of blood pressure, fluid status, and renal function is mandatory when renal function is compromised [9]. Accumulating evidence supports the administration of renin–angiotensin blockers in patients with proteinuria (>0.5 g/day) [69]. High-dose steroids and immunosuppressive agents such as azathioprine or mycophenolate mofetil and intravenous cyclophosphamide may be beneficial for moderate and severe diseases. Children with COVID-associated HSP may more frequently require the use of corticosteroids due to a possibly more severe disease course [72]. Similarly, adult and elderly patients should be treated more aggressively due to a more severe prognosis [73].

## 5. Conclusions

Despite the fact that HSP is the most prevalent, often self-limiting, systemic vasculitis in kids, its origin and pathophysiology are still unknown. About infectious agents, many viruses, including the SARS-CoV-2 virus, have been identified as the potential triggers of HSP with various hypothesized mechanisms. In addition, latent viral infections may reactivate during HSP, in particular following immunosuppressive therapies, complicating the complex clinical scenario of HSP. Furthermore, several vaccinations against viral infections, in particular recent COVID-19 vaccinations, have been associated with the development of HSP. Further studies are needed to increase our understanding of the role of viruses and vaccines in the etiology of HSP, a disease with a potentially severe clinical outcome.

## Data Availability

Not applicable.
